# Case Report: CMV-Associated Congenital Nephrotic Syndrome

**DOI:** 10.3389/fped.2020.580178

**Published:** 2020-11-27

**Authors:** Anju Jacob, Shameer M. Habeeb, Leal Herlitz, Eva Simkova, Jwan F. Shekhy, Alan Taylor, Walid Abuhammour, Ahmad Abou Tayoun, Martin Bitzan

**Affiliations:** ^1^Department of Pediatrics, Al Jalila Children's Specialty Hospital, Dubai, United Arab Emirates; ^2^Kidney Centre of Excellence, Al Jalila Children's Speciality Hospital, Dubai, United Arab Emirates; ^3^Department of Anatomic Pathology, Cleveland Clinic, Cleveland, OH, United States; ^4^Al Jalila Genomics Center, Al Jalila Children's Specialty Hospital, Dubai, United Arab Emirates; ^5^Section of Infectious Diseases, Al Jalila Children's Specialty Hospital, Dubai, United Arab Emirates; ^6^Department of Genetics, Mohammad Bin Rashid University of Medicine and Health Sciences, Dubai, United Arab Emirates

**Keywords:** Finnish-type nephrotic syndrome, *NPHS1*, cytomegalovirus, *Streptococcus pneumoniae*, case report, glomerulonephritis, infantile nephrotic syndrome

## Abstract

**Background:** Congenital nephrotic syndrome, historically defined by the onset of large proteinuria during the first 3 months of life, is a rare clinical disorder, generally with poor outcome. It is caused by pathogenic variants in genes associated with this syndrome or by fetal infections disrupting podocyte and/or glomerular basement membrane integrity. Here we describe an infant with congenital CMV infection and nephrotic syndrome that failed to respond to targeted antiviral therapy. Case and literature survey highlight the importance of the “tetrad” of clinical, virologic, histologic, and genetic workup to better understand the pathogenesis of CMV-associated congenital and infantile nephrotic syndromes.

**Case Presentation:** A male infant was referred at 9 weeks of life with progressive abdominal distention, scrotal edema, and vomiting. Pregnancy was complicated by oligohydramnios and pre-maturity (34 weeks). He was found to have nephrotic syndrome and anemia, normal platelet and white blood cell count, no splenomegaly, and no syndromic features. Diagnostic workup revealed active CMV infection (positive CMV IgM/PCR in plasma) and decreased C3 and C4. Maternal anti-CMV IgG was positive, IgM negative. Kidney biopsy demonstrated focal mesangial proliferative and sclerosing glomerulonephritis with few fibrocellular crescents, interstitial T- and B-lymphocyte infiltrates, and fibrosis/tubular atrophy. Immunofluorescence was negative. Electron microscopy showed diffuse podocyte effacement, but no cytomegalic inclusions or endothelial tubuloreticular arrays. After 4 weeks of treatment with valganciclovir, plasma and urine CMV PCR were negative, without improvement of the proteinuria. Unfortunately, the patient succumbed to fulminant pneumococcal infection at 7 months of age. Whole exome sequencing and targeted gene analysis identified a novel homozygous, pathogenic variant (2071+1G>T) in *NPHS1*.

**Literature Review and Discussion:** The role of CMV infection in isolated congenital nephrotic syndrome and the corresponding pathological changes are still debated. A search of the literature identified only three previous reports of infants with congenital nephrotic syndrome and evidence of CMV infection, who also underwent kidney biopsy and genetic studies.

**Conclusion:** Complete workup of congenital infections associated with nephrotic syndrome is warranted for a better understanding of their pathogenesis (“diagnostic triad” of viral, biopsy, and genetic studies). Molecular testing is essential for acute and long-term prognosis and treatment plan.

## Introduction

Congenital nephrotic syndrome (CNS) is a rare disease with poor renal and overall outcome. It is defined by the occurrence of large proteinuria and hypoproteinemia, resulting in generalized edema during the first 3 months of life (1). The estimated incidence is 1–3 per 100,000 children worldwide (2–4). The etiology of the CNS is heterogeneous and may present as part of a genetic syndrome.

Congenital infections, particularly CMV and *Treponema pallidum* (syphilis), and occasionally *Toxoplasma gondii*, and other pathogens, have long been associated with rare instances of CNS (5–12). Although CMV and cytomegalic inclusions have been demonstrated (predominantly) in renal tubular epithelial cells of patients with various CMV-associated glomerulopathies (7, 13), the causative role and the pathomechanism of the virus in cases of glomerulonephritis and nephrotic syndrome are still debated (4, 10, 14).

Recent surveys revealed the presence of disease causing genetic variants in up to 80% of CNS cases (4, 10, 15, 16). *Bona fide* pathogenic variants commonly lead to profound structural and functional abnormalities of the podocyte and/or glomerular basement membrane that compromise the glomerular filtration barrier. The prototypic, “Finnish type” congenital nephrotic syndrome is due to biallelic pathogenic variants in *NPHS1* (17, 18). Genes occasionally involved in congenital or infantile nephrotic syndrome with overlapping clinical and histological phenotypes include *NPHS2* (podocin), *LAMB2 (beta2-laminin)*, and *WT1* (Wilms Tumor 1 transcription factor), among others (4).

Early subtle histological alterations, such as microcystic proximal tubular dilatation, eventually lead to nephron loss and end stage kidney disease (ESKD) by the age of 2–3 years (1, 15, 19, 20). The spectrum of histological changes encompasses diffuse mesangial sclerosis (DMS), focal segmental glomerulosclerosis (FSGS), membranous glomerulopathy, and minimal change disease that are largely determined by the type of mutation and age at biopsy (1, 4, 15, 20, 21). The long-term management of patients with CNS remains challenging and may require early nephrectomy to minimize the pervasive effects of massive proteinuria, and subsequent kidney transplantation (4, 18, 22, 23).

We hypothesized that previously postulated infectious etiologies of CNS cases are biased due to the lack of biopsies and—historically not feasible—comprehensive genetic studies.

Here, we report an infant with CNS and CMV infection and demonstrate the importance of timely and complete diagnostic workup, i.e., the tetrad of clinical findings and virological/infectious disease, histopathologic, and molecular genetic studies.

## Case Description

A 9-week-old male (ex 34 weeks gestational age, corrected age 3 weeks) had been referred to our Emergency Department with suspected surgical abdomen. He presented a 3-day history of non-bilious, non-bloody vomiting and a 2-week-history of increasing abdominal distention and scrotal swelling. The patient was the second child of his parents who are from Kerala, India, and distantly related (3rd degree cousins). The couple's firstborn son is healthy.

Pregnancy was complicated by moderate oligohydramnios and decreased fetal movements which led to urgent C-section. Mother denied fever or rash during pregnancy, and her urinalysis was normal.

Birth weight was 1970 g (20th weight percentile). The weight of the placenta is not known. APGAR was 8 and 8 after 1 and 5 min, respectively. He received CPAP and then oxygen via nasal cannula for a total of 3 days. A post-natal brain ultrasound study was reportedly normal, TSH was 12 mU/L, and he was discharged home after 6 days. Immunizations were up-to-date, including PCV13.

### Clinical Exam

At presentation, the infant was alert, irritable, pale and grunting. He was normocephalic without dysmorphic features. There was no rash, no jaundice, no petechiae or ecchymoses. He was tachycardic at 175 beats per minute and tachypneic. The remainder of the cardiovascular and pulmonary findings was unremarkable. The abdomen was distended, tense and shiny with large ascites and no palpably enlarged liver or spleen. Substantial scrotal edema was noted. Weight (with edema) was 3.5 kg (0.01%, *z* score −3.83), length 52.5 cm (0.00%, *z* score −4.31), and head circumference 35.5 cm (0.04%, *z* score −3.35).

### Investigations

At the time of admission, there was large proteinuria, hypoalbuminemia, and hypercholesterolemia, consistent with nephrotic syndrome. Nephrotic range proteinuria was defined as a spot urine protein-to-creatinine ratio of >0.23 g/mmol (corresponding to >2.0 g protein/g creatinine). For details and definitions, see Table 1). The hemoglobin (Hb) level dropped from 100 to 69 g/L over the first 10 days of admission, with inadequate reticulocyte response and normal serum ferritin and iron levels. Serum C3 and C4 concentrations, measured on day 11 of admission, were decreased. The concomitant direct agglutination (Coombs) test was negative. Secondary findings were hypogammaglobulinemia, hypothyroidism with elevated TSH and low free T4 levels, hypovitaminosis D, low-normal serum ionized calcium (1.17 mmol/L) and moderately elevated intact PTH. ALT and AST were normal. GGT and bilirubin concentrations peaked during the first few days after admission (see Table 1).

**Table 1:** Laboratory results during the disease course^a^.

**Parameter**	**Reference ranges**	**Presentation (age 9 weeks)**	**Start VGC (age 10 weeks)**	**One month VGC (age 14 weeks)**	**Last results (age 7.4 months)**
Urine protein (g/L)	g/L	>2.0 (4+)	>2.0 (4+)	17.2	12.2
U protein creatinine ratio, nephrotic range	>0.23 g/mmol (>2.0 g/g)	>0.4 ^b^ (>3.52 g/g)	>0.7 ^b^ (>6.92 g/g)	6.44 (56.94 g/g)	3.71 (32.75 g/g)
Hematuria (dipstick/microscopy)	Dipstick negative 0–3/HPF	Small -	Small -	3+ >35	3+ >35
Serum albumin	33–54 g/L	9	17 ^c^	9	12
Total serum protein	51–73 g/L	24.5	33.2	25.5	-
Toxoplasma IgM/IgG		neg/neg	-	-	-
Rubella IgM/IgG		neg/neg	-	-	-
CMV IgM	<0.69 COI	4.56	-	-	-
CMV IgG	<0.49 U/mL	5.54	-	-	-
CMV PCR blood		positive	-	negative	-
CMV PCR urine		positive	-	negative	-
HSV 1 & 2 PCR blood		neg/neg	-	-	-
Syphilis (*T. pallidum*)		Neg	-	-	-
Hepatitis B (HBs) Ag	<1.0 COI	-	-	0.394	-
Anti-HBs Ab	<10 mIU/mL	<2.00	-	4.2 ^d^	-
Anti-HBc Ab		Non-reactive	-	-	-
Hepatitis C (anti-HCV Ab)		Non-reactive	-	-	-
EBV PCR (blood)		negative	-	-	-
Hemoglobin	111–131 g/L	100 ^e^	69 ^f^	93	106
WBC	6.0–16.0 × 10^9^/L	11.7	12.6	5.6	16.8
Absolute neutrophil count	1.00–6.00 × 10^9^/L	1.62	0.79	0.79 ^g^	4.8
Platelets	200–550 × 10^9^/L	328	357	584 ^h^	827
Serum IgG	3.09–15.73 g/L	<0.4	-	-	-
Serum IgA	0.08–0.58 g/L	0.11	-	-	-
Serum IgM	0.04–0.89 g/L	0.49	-	-	-
Serum C3	0.82–1.67 g/L	-	0.31	-	-
Serum C4	0.14–0.44 g/L	-	0.08	-	-
C-reactive protein (CRP)	0–2.80 mg/L	0.42	-	0.63	-
Cholesterol	2.1–3.8 mmol/L	10.4	-	-	10.4 ^i^
ALT	0–57 U/L	17	11	12	10 ^i^
AST	0–110 U/L	28	40	27	30 ^i^
GGT	0–203 U/L	1036/519 ^j^	226	161	61 ^i^
Total bilirubin	0–20.52 μmol/L	9.75/41.55 ^j^	13.68	<2.39	<2.39 ^i^
25 OH D3	75–250 nmol/L	32.5	-	-	27.5 ^l^
1,25 (OH)_2_ D3	48–190 pmol/L	-	-	-	221 ^I^
Intact PTH	1.6–6.7 pmol/L	10.8	-	-	11.3 ^l^
TSH	0.73–8.35 mU/L	23.23	4.97 ^k^	-	4.11 ^m^
Free T4	11.9–25.6 pmol/L	7.1	22.2 ^k^	-	12.4 ^m^
Body weight	kg	3.50 ^n^	3.20	4.05	5.22
WHO growth chart	Centile (z score)	0.01 (−3.83)	0.00 (−4.89)	0.00 (−4.01)	0.00 (−4.21)
Head circumference	cm	35.5	-	-	-
WHO growth chart	Centile (z score)	0.04 (−3.35)	-	-	-

a *COI cut-off index, HPF high power field, VGC valganciclovir, - not obtained at that indicated interval*.

b *Protein titration in urine was not available initially. See Kidney Disease Improving Global Outcomes (KDIGO) Clinical Practice Guideline for Glomerulonephritis (2012) for the definition of nephrotic-range proteinuria*.

c *Following albumin infusions*.

d *Measured 2 weeks after commencing after HBV vaccination*.

e *Normocytic anemia with inadequately low reticulocyte count (2.38%)*.

f *Patient received RBC transfusion following this result*.

g *Subsequent neutrophil counts remained >2 × 10^9/L^*.

h *Persistently elevated platelet counts following treatment with VGC and the suppression of detectable CMV DNA*.

i *Last measurement at age 5.1 months*.

j *Change from day 1 to day 3 of admission; direct bilirubin was about 20% of total bilirubin*.

k *Rise of serum bilirubin concentration prior to commencement of VGC treatment*.

l *Supplementation with 2,000 IU cholecalciferol daily*.

m *Measurements during L-thyroxin supplementation*.

n *edematous*.

The ultrasound (US) scan showed enlarged kindeys with mildly increased cortical echogenicity. Liver and spleen were of normal size and echotexture. The brain US study was normal, except the presence of thalamostriate mineralizing vasculopathy.

Cardiac echography demonstrated a small perimembranous ventricular septal defect of 2–3 mm with left-to-right shunt and patent foramen ovale, none requiring specific interventions.

Infectious disease workup revealed anti-CMV IgM antibodies and CMV DNA (by PCR) in blood and urine. Maternal screening during pregnancy for toxoplasma (IgG and IgM), rubella (IgM), HIV 1 & 2 antibodies/P24 antigen, hepatitis B (HBsAg) and hepatitis C (antibodies), and syphilis was negative. Subsequent CMV testing showed high maternal serum concentrations of CMV IgG, but no anti-CMV IgM.

### Treatment of the Patient

Treatment consisted of frequent albumin infusions, diuretics and ACE inhibition, with improvement of ascites and peripheral edema. He also received L-thyroxin, vitamin D and oral penicillin prophylaxis, low-dose acetyl salicylic acid, iron, and indomethacin. In addition, he was vaccinated against *Streptococcus pneumoniae* (PCV13, twice) and hepatitis B.

Valganciclovir treatment was initiated 9 days after presentation at a dose of 22 mg/kg/day. CMV DNA became undetectable in plasma and urine after 1 month of antiviral therapy, however proteinuria failed to improve (Table 1) strengthening the presumptive clinical diagnosis of a genetic form of congenital nephrotic syndrome.

The patient's energy and protein intake remained precariously inadequate. However, parents were reluctant to agree to g-tube insertion or (unilateral) nephrectomy. Tragically, at the age of 7.9 months, following a few days of lapsed penicillin administration due to vomiting and diarrhea, the patient succumbed to fulminant sepsis, within hours after arrival in the Emergency Department, caused by pan-sensitive *S. pneumoniae*.

### Kidney Biopsy

**Figure 1:**
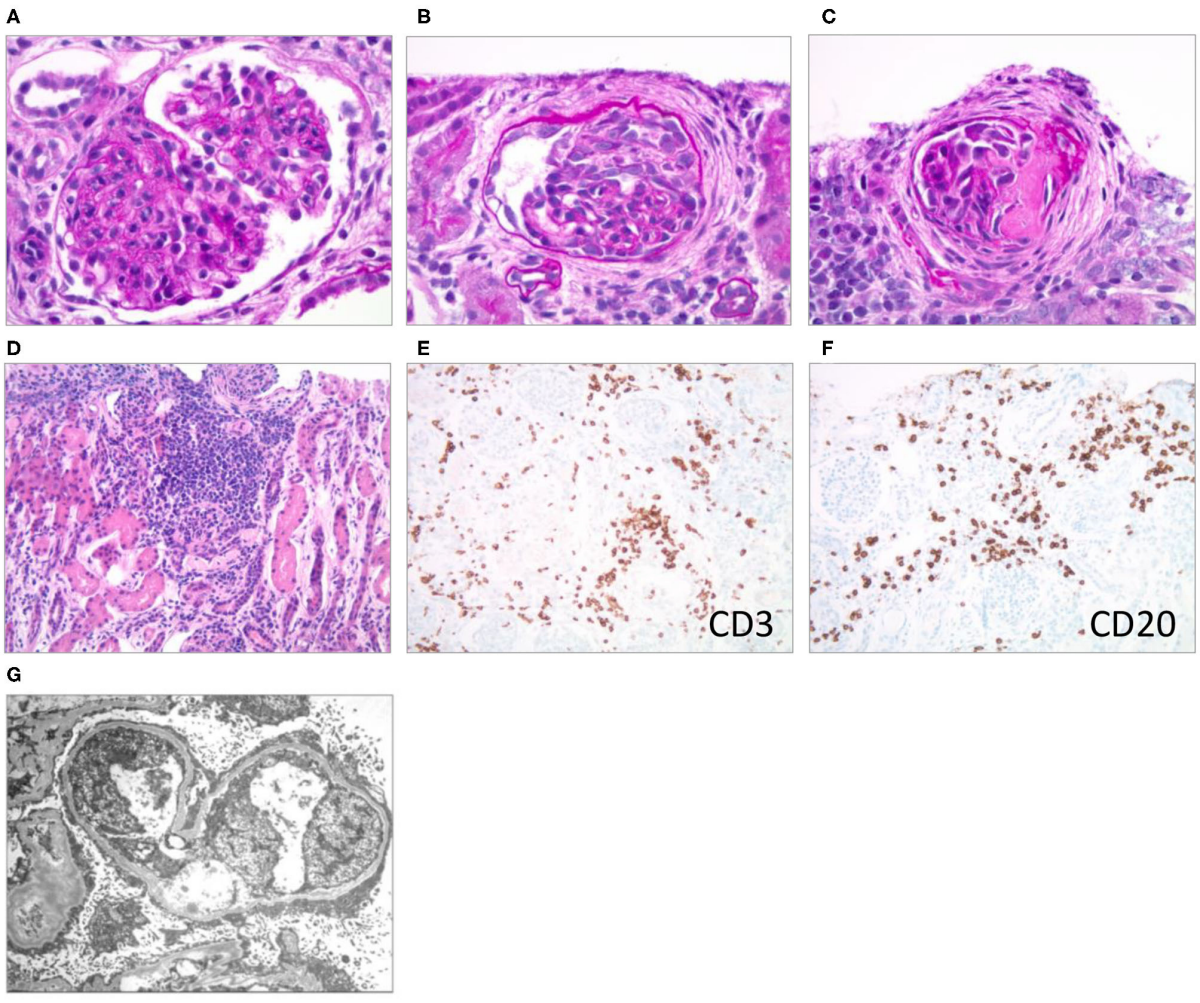
Renal pathological presentation (kidney biopsy of the proband 15 days after admission). **(A–C)** Brightfield microscopy (PAS stain x600); **(D)** brightfield (H & E stain x200); **(E,F)** immunohistochemistry (x200); **(G)** electron microscopy. **(A)** Mesangial and endocapillary hypercellularity, **(B)** cellular crescent, **(C)** segmental scar/fibrous crescent, **(D–F)** interstitial infiltrates, **(G)** diffuse podocyte foot process effacement with prominent microvillous transformation.

A kidney biopsy was performed 15 days after hospitalization (6 days after initiation of VGC treatment) to differentiate the pathohistological changes underlying the nephrotic presentation (1, 2, 4, 12). The majority of the >100 sampled glomeruli showed mild mesangial hypercellularity with patent capillaries and normal glomerular basement membrane (GBM) contours. Ten percent of the glomeruli were globally sclerosed or segmentally scarred. Three glomeruli showed active cellular or fibrocellular crescents with proliferation in the Bowman space and focal ruptures of GBM. There was a patchy, mononuclear tubulointerstitial inflammatory infiltrate. Infiltrating interstitial lymphocytes stained positive for CD3 and CD20, respectively, indicating the presence of T- and B-cells. Trichrome staining showed fibrosis in <5% of the cortex. Arteries and arterioles were histologically normal. Immunohistochemical staining for CMV was negative. Routine immunofluorescence showed faint, likely non-specific staining for IgM and C3. Electron microscopy revealed diffuse podocyte foot process effacement with prominent microvillous transformation, but no immune-type deposits. Endothelial fenestrations were intact, and no viral or endothelial tubuloreticular inclusions were noted (Figure 1).

### Genetic Studies

Genomic DNA, extracted from peripheral blood cells, underwent a series of ultra-sonication, chemical, and enzymatic steps to generate a sequencing-ready library of short fragments (300–400 bp) using the SureSelect^XT^ kit (Agilent, USA). RNA capture probes targeting all coding regions were used to enrich for whole exome regions using the SureSelect Clinical Research Exome V2 kit (Agilent, USA). The enriched library was then subjected to next generation sequencing (2 × 150 bp) using the SP flow cell and the NovaSeq platform (Illumina, USA). Sequencing data were then processed using an in-house custom made bioinformatics pipeline to retain high quality sequencing reads with at least 100X coverage across all coding regions. Rare variants in 99 genes associated with nephrotic syndrome (Supplementary Table 1) were filtered for analysis and interpretation using the American College of Medical Genetics and Genomics (ACMGG) Sequence variant interpretation guideline (24). Two variants in the *NPHS1* and the *ITSN2* genes met the ACMGG criteria for reporting. *NPHS1* encodes nephrin which is central for the integrity of the podocyte slit diaphragm. Pathogenic variants in *NPHS1* cause classical Finnish-type congenital nephrotic syndrome (17). The apparently homozygous 2071+1G>T variant in *NPHS1* (NM_004646.3) has not been previously reported in individuals with disease and is absent from large population studies such as the Genome Aggregation Database (genomAD) and the Greater Middle East (GME) Variome database. This variant occurs in the conserved region (±1,2) of the splice consensus sequence of the only known *NPHS1* transcript, and is predicted to cause altered splicing leading to an abnormal or absent protein (25). The mutation is apparently homozygous, although a large deletion of the second allele could not be ruled out since parental testing was not conducted.

A heterozygous c.2713G>A p. (Ala905Thr) missense variant was also reported in *ITSN2* (NM_006277.2), the gene encoding intersectin 2, a member of the guanine exchange factor (GEF) family of proteins that activate Cdc42. This variant was classified as uncertain due to lack of sufficient evidence supporting its clinical significance. Bi-allelic pathogenic variants in *ITSN2* have been reported as a novel cause of nephrotic syndrome (26). The identified variant is absent from large population studies such as the Genome Aggregation Database (gnomAD) and the Greater Middle East (GME) Variome database. Computational prediction tools did not provide evidence for or against pathogenicity. No variants were discovered in complement-related genes or genes associated with (other) immunodeficiencies.

## Survey of the Literature

### CMV-Associated Congenital and Infantile Nephrotic Syndrome

Following the identification of a pathogenic *NPHS1* variant as the genetic cause of the patient's nephrotic syndrome, we wondered whether recognizable histopathological features would allow differentiating CMV-associated lesions (and CMV CNS) from CNS due to defined genetic variants. We therefore searched the available literature (PubMed and Google Scholar, without language restriction) for CMV-associated/infantile nephrotic syndrome with histological and genetic findings (“tetrad” of documented CMV infection and clinical features, kidney biopsy, and mutation screen).

Our survey identified only three cases with a complete tetrad: one patient with a homozygous *NPHS2* pathogenic variant, one patient with reported pathogenic variants in both *NPHS1* and *COL4A5* (however the number of variants and the phase of the variants in each gene was not detailed), and one with no detectable variants in *NPH1, NPH2*, and *WT1* (Table 2; patients #3, 7, and 6, respectively) (12, 14, 31). All three patients were treated with ganciclovir (GCV). Only patient #6 achieved sustained remission of proteinuria (31).

**Table 2:** CMV-associated congenital and infantile nephrotic syndrome (Literature review and proband).

**#**	**Age onset Sex (gestation)**	**Pregnancy/ perinatal**	**Clinical presentation**	**Urinalysis Serum albumin**	**Infectious diagnostic**	**Treatment**	**Proteinuria after antiviral**	**Outcome**	**Genetic variants**	**References**
1	5 mo F (39 w)	PBWR 0.2 (Maternal NS 8 mo in pregnancy (FSGS), resistant to Pred & POCY)	Normal at birth At 5 mo generalized edema At 6.5 mo fever, purpuric maculo-papular rash, hepatomegaly elevated ALT, LDH	Large proteinuria (>40 mg/m^2^/h) Hypoalbuminemia (23 g/L)	At 5 mo CMV IgM+ (mat CMV IgM-, IgG+) (both Toxo, HSV, VZV, RV, Syph, HBV, HCV neg) (C3 &C4 N) At 6.5 mo CMV B-, U+, IgM+	At 5mo: High-dose Pred (ineffective) At 6.5mo GCV x 15 d	**Resolved** (proteinuria improved at 15d)	At 12 mo Clinically well, all symptoms resolved Sustained proteinuria remission, N renal function	ND	(27)
2	5 mo M (Term)	PBWR 0.19	Acute, generalized edema after URTI/vomiting US KUB normal	Large proteinuria (>40 mg/m^2^/h) Hypoalbuminemia (6.9 g/L)	CMV IgM+, IgG+ (EBV, HBV/HBC, HIV, HSV, Syph, VZV neg) (mat CMV IgM-, IgG+)	Pred 60 x4w (ineffective), then oral GCV x12w	**Resolved** (remission at 4 w GCV)	Sustained proteinuria remission Stable at 17 mo FU	ND	(28)
3	45 d F (36 w)	Mild dysmorphic features Antenatal US large, echogenic kidneys) Ebstein anomaly	Generalized edema/ascites/pleural effusion Bilateral vitreitis Bilateral SNHL Increased renal echogenicity	Large proteinuria (170 mg/m^2^/h) Hypoalbuminemia (22 g/L)	CMV U+, IgM+ (Toxo, RV, Syph, HBV, HCV, HIV neg) C normal	Daily IV Alb/ diuretic GCV (duration NR)	Persistent	Resolution SNHL & vitreitis 6 mo post-GCV ESRD at 21 mo	*NPHS2* ^**a**^	(14)
4	57 d F (33 w)	Pregnancy uncomplicated Not dysmorphic	Generalized edema, HTN Respiratory symptoms Anemia 58 g/L, platelets 75/nL Enlarged, echogenic kidneys Brain cortical atrophy (no calcifications, no retinitis)	Large proteinuria (454 mg/m^2^/h) Hypoalbuminemia (20 g/L) UA: WBC, RBC, gran casts	CMV IgG+, IgM- CMV PCR serum high (neg HBV/HCV, HSV1/2, RV, toxo Ab) (Mat CMV IgM-, IgG+)	Daily IV Alb & diuretics Captopril IV GCV x 3 w	**Resolved** Upc 0.079 g/mmol at 3w	Hematologic response, HTN resolved Remission >14 mo FU	ND	(29)
5	6 mo	NR	Persistent watery diarrhea Generalized edema & ascites Hb 107 g/L Plt 406/nL	Large proteinuria (28 g/L) Hypoalbuminemia (10 g/L)	CMV PCR (blood/urine?)	IV alb x 19d Captopril IV GCV x 2w, then VGC x 4w	**Resolved** Up low (CMV PCR neg) at 19d GCV/VGC	Resolution of colitis CMV PCR neg 19d Stable remission 1y	ND	(30)
6	5 mo M	Diamniotic twin pregnancy	Gastroenteritis Mild edema Large kidneys No dysmorphism/brain lesions Normal ophthalmological exam	Large proteinuria (Upc 7.83 g/mmol) Hypoalbuminemia (10.3 g/L) Normal GFR	CMV B-, PCR+ (EBV (PCR), HBV, HCV, HIV, Syph neg)	IV Alb x 16d Captopril x 16d IV GCV x 15d (from D15), then VGC x 15d	**Resolved** Up 0.15 g/L at 15d, Salb N	Sustained remission over 30 mo FU	Negative (*NPHS1, NPHS2, WT1*) ^b^	(31)
7	15 d	NR	“asymptomatic”	Large proteinuria (3+, 2.82 g/mmol) Hypoalbuminemia (21.7 –>11.0 g/L) Normal cholesterol	CMV IgM+	GCV 10 x 4w	Partial remission (Upc 0.189, S-Alb 30.4 g/L)	CKD at 13mo (eGFR 55 mL/min/1.73m^2^) Proteinuria 3+	*NPHS1, COL4A5* ^c^	(12)
8	51 d M (34 w)	Oligohydramnios asymmetric IUGR	Ascites, scrotal edema FFT, developmental delay Anemia (100 69 g/L) GGT/mild hyperbilirubinemia Thalamostriate mineralizing vasculopathy	Large proteinuria (4+, 3.4 g/mmol) Microhematuria (15-20 RBC/HPF) Hypoalbuminemia (9 g/L) eGFR normal	CMV IgM+ CMV B-PCR+ CMV U-PCR+ (Toxo, RV IgM, Syph neg) Mat CMV IgG+, IgM- Low C3 &C4	Captopril VGC IV Alb/diuretic Pen-VK prophylaxis	persistent	CMV PCR neg 4 w post VGC, FFT Death pneumococcal sepsis at 7.9 mo	*NPHS1 (INTS2 – het)* ^d^	This report

a *Homozygous for nonsense mutation c.412C < T (p.Arg138X), carrier status confirmed for both parents (14, 32)*.

b *Testing was limited to the indicated genes. Methodological details are not reported*.

c *NPHS1 variant reported as c.2396G>T (p. Gly799Val) and c.1339G>A (p. Glu477Lys), COL4A5 variant not specified (12)*.

d *Detail see Results section*.

*CKD, chronic kidney disease; d, day(s); eGFR, estimated glomerular filtration rate (33); ESRD, end stage renal disease; FFT, failure to thrive; GGT, gamma glutamyl transferase; GCV, ganciclovir (IV); Hb, hemoglobin; HPF, high power field; HTN, hypertension, mo month(s); N, normal; NR, not reported; PBWR, placenta birthweight ratio; Plt, platelet(s); Upc, (urine protein-to-creatinine ratio), VGC, valganciclovir (oral); w, week(s)*.

*SI unit conversion Upc (g/mmol) × 8.84 = g/g (reference range for nephrotic range proteinuria >0.23 g/mmol, corresponding to 2.0 g/g)*.

We then searched for reports of patients with CMV-associated congenital or infantile nephrotic syndrome, who also had a kidney biopsy. All identified cases (#1, 2, 4, 5) were treated with GCV (or GCV, followed by VGC), and all entered stable, proteinuria-free remission (Table 2). Mean antiviral treatment duration was 38 ± 27 (median 30) days; proteinuria improved substantially or disappeared after a mean of 20 ± 5 (median 19) days. The nephrotic syndrome was GCV-resistant in three cases (#3, 7, and 8). These patients were significantly younger at nephrotic syndrome onset (median 1.5, range 0.5–1.7 months) than GCV “sensitive” patients (median 5, range 1.9–6 months) (*P* < 0.05, unpaired *t*-test). There was no apparent difference in the presenting nephrotic features (urine protein excretion and serum albumin concentrations) between GCV/VGC responsive and refractory patients.

### Kidney Biopsy Results in the Literature

Results of the biopsy survey are detailed in Table 3. In this analysis we included also patients without genetic results under the assumption that stable remission from CMV-associated nephrotic syndrome indicates the absence of podocyte gene mutations. We identified and evaluated eight patients (including our case) with a total of 9 biopsies. The biopsies of four of the five GCV “sensitive” (mutation “negative”) patients (#1, 2, 4, and 6) (27–29, 31) revealed mild-moderate mesangial cell proliferation with or without (mesangial) matrix increase. One of these biopsies also demonstrated mesangial sclerosis (#4). This contrasts with the findings in patients with GCV “resistant” (mutation “positive”) CNS (#3, 7, and 8) (12, 14), where light microscopic findings demonstrate a spectrum of normal-appearing glomeruli (#3a), glomerular sclerosis (#3b and 8) and occasional cellular or fibrocellular crescents, as well as focal mesangial proliferation (#7 and 8).

**Table 3:** Renal biopsy findings (Literature review and proband).

**#**	**Onset/Bx (age in mo)**	**Glomerular compartment/BFM**	**IF/IH**	**EM**	**Tubulo-interstitial compartment**	**CMV-related findings**	**Genetic findings^**a**^**	**References**
1	5/5.5	Enlarged glomeruli Mild mesangial cell proliferation	minimal mesangial IgM & C1q	Podocyte vacuolization w/o enlargement Foot process effacement Normal GBM Minor mesangial deposits Enlarged endothelial cells/single platelet thrombus/ scattered fibrin strands	NR	Endothelial tubulo-reticular arrays	ND	(27)
2	5/5	Mod mes cell & matrix increase in 25% of glomeruli w/o sclerosis	Diffuse mes IgM 2+	GBM normal	Normal w/o infiltrates or atrophy	No CMV inclusions Tissue PCR CMV+	ND	(28)
3a	1.5/ <2	Normal-appearing glomeruli	NR/normal	NR	Normal (and normal vasculature)	Single inclusion body in distal tubule	*NPHS2*	(14)
3b	1.5/16	3/13 glomeruli globally sclerosed Remainder w/ mild mesangial proliferation & expansion	NR/normal	Foot process effacement	Normal (and normal vasculature)	Inclusion bodies absent Tissue CMV PCR & culture neg		
4	2/~2.3	Increased mes matrix & cell proliferation Some glomeruli w/ global, the remainder w/ seg mes sclerosis	Absent staining for IGs & C	NR	Focal tubular atrophy and fibrosis w/ tubular dilatation. Some tubules w/ Inflammatory infiltrates	Cytomegalic inclusion bodies in tubules and some glomeruli	ND	(29)
5	6/6	Mild endocapillary proliferation ^b^	NR	NR	Atypical heavy tubular lesions, interstitial edema	NR	ND	(32)
6	5/5	Mild mes cell hypertrophy No mes sclerosis	NR	ND	Non-specific tubular lesions Interstitial edema	Absent viral cytopathic inclusions	Negative	(31)
7	0.5/2	Mild mesangial proliferation and Matrix increase. Multiple immature glomeruli with cellular crescents	Mesangial deposits of IgM (++) and C3 (+)	Mild mesangial cell/ matrix hyperplasia Podocyte foot process fusion Normal GBM No electron dense deposits	Vacuolar & granular degeneration tub epithelial cells, small focal atrophy Focal lymphoid interstitial mononuclear cells Eosinophil infiltrates w/ fibrosis Thickened small arterial walls	No CMV inclusions, CMV DNA neg	*NPHS1 COL4A5*	(12)
8	1.7/2.6	Global or segmental sclerosis in 10% Focal mesangial proliferation Few cellular & fibrocellular crescents Mild cortical fibrosis (<5%)	Negative (non-specific trapping of IgM and C3)	Diffuse podocyte foot process effacement w/ microvillous transformation Intact endothelial fenestration	IF/TA Patchy, moderate tubule-interstitial infiltrates (CD3+, CD20+)	No endothelial tubulo-reticular arrays Staining for CMV negative	*NPHS1*	This report

a *Homozygous or compound heterozygous (except for COL4A5). For genetic details, see Table 2*.

b *Very brief pathology description*.

*BFM, brightfield microscopy; Bx, kidney biopsy; C, complement; CKD, chronic kidney disease; d, day(s); EM, electron microscopy; ESRD, end stage renal disease; GCV, ganciclovir (IV); IF/IH, immunofluorescence/immuno histological stain; IF/TA, interstitial fibrosis/tubular atrophy; IG(s), immunoglobulin(s); mes, mesangial; mo, month(s); ND, not done; neg, negative; NR, not reported; w, week(s)*.

## Discussion

The presented case demonstrates features of classical (Finnish-type) congenital nephrotic syndrome (CNS) due to a homozygous *NPHS1* pathogenic variant, complicated by congenital CMV infection. There was no retinopathy or microcephaly, and apart from (non-specific) thalamostriate mineralizing vasculopathy (34, 35), the infant had no detectable cerebral abnormalities by ultrasound. However, he had substantial anemia (resulting in RBC transfusion), neutropenia (without thrombocytopenia) and temporary GGT and bilirubin elevation (without hepato- or splenomegaly). The C3 and C4 hypocomplementemia was surprising and deserves further inquiry in comparable cases of congenital/Finnish type nephrotic syndrome and of congenital CMV infections, whereas the extreme hypogammaglobulinemia resulted in all likelihood from massive protein loss via the disrupted glomerular filtration barrier.

Intrauterine or perinatal CMV exposure may have been responsible, at least in part, for the presenting signs and symptoms in our patient. However, there are no criteria to predict the role of CMV in this or similar patients' nephrotic syndromes. Due to health insurance reasons, we were unable to pursue early genetic testing. However, we proceeded with a kidney biopsy for diagnostic and therapeutic guidance (1, 4).

The histological findings were consistent with what has been described in CMV-related congenital nephrotic syndrome, including proliferative lesions and diffuse podocyte effacement. Lack of CMV detection (or inclusion bodies) in renal tissue makes it less likely that the virus was the direct cause of the observed morphological changes. Some of the demonstrated lesions may have arisen as part of the inflammatory and cytokine response of the host, albeit without virus or interferon-gamma-induced (endothelial) tubuloreticular arrays (36, 37). Interestingly, serum C3 and C4 protein levels were decreased during the early course, yet we failed to observe unequivocal immune deposits in the kidney biopsy.

Antiviral treatment was combined with supportive measures, mainly diuretic-assisted albumin infusions, thyroid hormone and vitamin D which controlled the profound edema and hormonal deficiencies. Persistence of large proteinuria and dependency on albumin infusions after the CMV PCR had become negative, favored a genetic cause of the nephrotic syndrome. Attempts to convince the parents to optimize nutrition (g-tube feeding) and (unilateral) nephrectomy to reduce protein losses (2, 4, 22) remained unsuccessful. Unfortunately, he succumbed to foudroyant pneumococcal sepsis at almost 8 months of age, despite repeat vaccination against *S. pneumoniae* and the prescription of antibiotic prophylaxis, which highlights the highly immunocompromised status of infants with severe nephrotic syndrome. We were eventually able to perform whole exome sequencing and can at least offer genetic counseling for the family.

The occurrence of NS in newborns with intrauterine exposure to CMV has been known since more than 50 years (7, 13). While infants with overwhelming congenital CMV disease demonstrate CMV invasion and proliferation in the kidney along with many other tissues (7), the mechanism of CMV-induced glomerular injury and podocytopathy is not well-defined. Previous studies have addressed direct, virus-mediated tissue injury and injury induced by the host immune response, such as T cell infiltration or immune complex formation (38, 39). However, the natural history and outcome of theses lesions has not been adequately documented, and there is a paucity of cases correlating virological and serological results with renal tissue and genetic studies.

The historical attribution of (severe) CNS to concurrent (congenital) CMV infection, including some of the histological changes, needs be revisited (4, 14). This notion does not negate the possible occurrence of (large) proteinuria and edema in newborns with severe, CMV-induced injuries, such as microcephaly or sensorineural hearing loss, with or without hepato- and splenomegaly and cytopenia.

Although the number of reported cases of CMV-associated CNS with a complete diagnostic “tetrad” is surprisingly small (Table 2), our analysis seems to confirm that an onset of nephrotic syndrome within 3 months of life predicts an underlying variant within podocyte or GBM-related genes, regardless of a coincidental CMV infection. In contrast, onset of nephrotic syndrome between 4 and 12 months and active CMV proliferation in previously asymptomatic infants appears to increase the likelihood of CMV as the remediable cause of the nephrotic syndrome. Neither the severity of proteinuria or hypoalbuminemia at presentation, nor (estimated) GFR or hematological parameters (anemia or thrombocytopenia)–where reported–appear to have discriminatory power in this small cohort.

Table 3 juxtaposes the histological findings of CMV-associated congenital and infantile NS. All but one patient demonstrating sustained remission of proteinuria following antiviral therapy, presented after 3 months of age. One of the common features of all patients with CMV-associated congenital or infantile nephrotic syndrome appears to be (usually mild) mesangial proliferation with or without matrix accumulation. Segmental and global glomerulosclerosis (40) and crescent formation is only noted in the two *NPHS1* patients. Given the paucity of completely defined cases and the heterogeneity of renal pathology among patients with autosomal recessive forms of CNS, the histological differentiation between CMV-induced congenital or infantile NS and patients with an underlying genetic variant remains challenging.

The *NPHS1* mutation identified in our patient is apparently homozygous, although a large deletion of the second allele could not be ruled out as parental testing was not possible. The recurrence risk would remain the same [25%] if the variant was homozygous, or compound heterozygous with a pathogenic deletion. Identifying the exact variant(s) allows for targeted variant analysis either prenatally or via *in vitro* fertilization and pre-implantation genetic diagnosis) for any future pregnancies.

The strength of this report is the complete characterization of the case and others identified in a comprehensive literature survey. Its limitation—and reason for publication—is the small number of fully described cases.

## Conclusions

Genetic testing is warranted in all patients presenting with isolated or syndromic nephrotic syndrome immediately after birth or within the first 2–3 months of life, whether CMV is present or not. To further clarify the specific contributions of CMV and CMV disease to the histological changes in the kidney and severe, persistent nephrotic syndrome, a correlation (tetrad) of clinical and laboratory findings, kidney biopsy with all staining modalities and/or molecular tools, and genetic work-up are needed.

## Ethics Statement

Written informed consent was obtained from the minors' legal guardian/next of kin for the publication of any potentially identifiable images or data included in this article.

## Author Contributions

AJ collected the data, reviewed the literature, and wrote the first draft of the manuscript. SH, ES, JFS, and AJ participated in the care of the patient. LH read and interpreted the kidney biopsy and provided the photographs. AT and AA were responsible for genetic counseling, performed whole exome sequencing, and analyzed and interpreted the molecular data. WA provided expert infectious disease consultation. MB supervised the care of the patient, reviewed all clinical and laboratory data, performed an independent comprehensive literature review, and wrote the final version of the manuscript. All authors critically reviewed and edited the manuscript.

## Conflict of Interest

The authors declare that the research was conducted in the absence of any commercial or financial relationships that could be construed as a potential conflict of interest.
