# Supplementary material for: Case Report: CMV-Associated Congenital Nephrotic Syndrome
Source: Front Pediatr. 2020 Nov 27;8:580178. doi: 10.3389/fped.2020.580178 (PMC7728737; doi:10.3389/fped.2020.580178)
Supplement: Supplementary file 1 [file Table_1.DOCX]

**Supplementary Table S1.** Gene list (99) hereditary nephrotic syndrome

*ACTN4, ALG1, ALMS1, ANKFY1, ANKS6, ANLN, APOL1, ARHGAP24, ARHGDIA, CD151, CD2AP, CDK20, CFH, CLCN5, COL4A1, COL4A3, COL4A4, COL4A5, COQ2, COQ6, COQ7, COQ8B, COQ9, CRB2, CUBN, CYP11B2, DGKE, DLC1, E2F3, EMP2, FAT1, GAPVD1, GLA, GPC5, INF2, ITGA3, ITGB4, ITSN1, ITSN2, KANK1, KANK2, KANK4, KIRREL1, LAGE3, LAMB2, LMNA, LMX1B, MAFB, MAGI2, MED28, MEFV, MUC1, MYH9, MYO1E, NEIL1, NPHP4, NPHS1, NPHS2, NUP107, NUP133, NUP160, NUP205, NUP85, NUP93, NXF5, OCRL, OSGEP, PAX2, PDSS2, PLCE1, PMM2, PODXL, PTPRO, SCARB2, SMARCAL1, SYNPO, TBC1D8B, TNS2, TP53RK, TPRKB, TRPC6, TTC21B, VIPAS39, VPS33B, WDR73, WT1, XPO5, ZMPSTE24, AGXT, FN1, GSN, NPHP1, PLCG2, SGPL1, WDR4, NEU1, GON7, NFKB2, YRDC*
